# Case Report: Unraveling clinical heterogeneity in DEPDC5-related epilepsy: a genotype–phenotype correlation study in eight pediatric cases

**DOI:** 10.3389/fnins.2025.1595667

**Published:** 2025-10-09

**Authors:** Tong Zhao, Fang Chen, Bin Cao, Liqun Yang, Jiaqi Yin, Yanqi Li, Fan Feng

**Affiliations:** ^1^Department of Neurology, Hebei Children's Hospital, Shijiazhuang, Hebei, China; ^2^Trauma Center of Shijiazhuang People's Hospital, Shijiazhuang, Hebei, China; ^3^Department of Endocrinology, Hebei General Hospital, Shijiazhuang, Hebei, China

**Keywords:** epilepsy, gene mutation, inheritance, DEPDC5, phenotype

## Abstract

**Objective:**

To summarize the clinical characteristics of eight children with DEPDC5 gene variant-associated epilepsy.

**Methods:**

Clinical data of children with DEPDC5-related epilepsy treated at Hebei Provincial Children’s Hospital from April 2020 to November 2024 were retrospectively analyzed.

**Results:**

Among the 8 children (5 males, 3 females), age of onset ranged from 1 year 4 months to 9 years 3 months. Genetic analysis revealed 6 missense mutations, 1 code-shift mutation, and 1 large segment deletion, including 1 *de novo* and 7 hereditary mutations. Four children exhibited global developmental delay. Seizure types included generalized tonic–clonic (5 cases), tonic (1 case), and 2 forms of seizures: tonic seizures and atonic seizures (2 cases). EEG showed abnormal discharges in all cases: focal (4 cases), multifocal (3 cases), and slow-wave (1 case). Brain MRI abnormalities were observed in 4 children, including delayed myelination, hyaloid septal cavities, and microgyrus malformation. Diagnoses included Lennox–Gastaut syndrome (4 cases) and self-limiting epilepsy with centrotemporal spikes (2 cases). Six children responded well to medication (seizure reduction >25%), while 2 had poor control.

**Conclusion:**

DEPDC5 gene mutations result in diverse phenotypes, potentially influenced by age of onset and mutation type. Generalized seizures were most common, with high rates of EEG abnormalities and structural brain changes. In a few cases (3 cases), treatment with levetiracetam and phenobarbital can reduce the frequency of epileptic seizures by 25%, but due to limited sample size, its exact efficacy still needs further research and verification.

## 1 Introduction

The DEPDC5 gene is located on chromosome 22 at the q12.2-q12.3 region, comprising 43 exons that encode 1,603 amino acids. The DEPDC5 protein encoded by this gene is a GTPase-activating protein (GAP) containing five domains: N-terminal, SABA, SHEN, DEP, and C-terminal. It is constitutively expressed in brain cells during early embryonic development (Samanta, 2022). The DEPDC5 protein forms the GATOR1 complex with NPRL2 and NPRL3 proteins, functioning to regulate cellular growth, proliferation, and metabolism by modulating the mTOR (mammalian target of rapamycin) signaling pathway. This protein complex primarily regulates the mTOR pathway by modulating RAG GTPase activity through changes in amino acid levels. Elevated amino acid levels activate RAG GTPase activity, thereby activating the mTOR pathway (Marsan et al., 2016). It has been discovered that mutations in the DEPDC5 gene disrupt the function of the DEPDC5 protein it encodes. As the core component of the GATOR1 complex, impaired DEPDC5 ultimately disrupts mTOR signaling, leading to neuronal dysfunction and structural abnormalities in brain development. These alterations are closely associated with a range of diseases including epilepsy and cancer (Tokorodani et al., 2024).

Diseases associated with DEPDC5 gene variants exhibit complex genetic and phenotypic characteristics. The primary inheritance pattern is autosomal dominant germline mutations. However, it is noteworthy that some patients demonstrate somatic mutation mechanisms, where mutations occur during embryonic development and are confined to specific cells within localized brain regions (Ferri et al., 2017; French et al., 2016), explaining why some patients without a family history exhibit focal brain lesions. Clinically, the disorder exhibits two key features: incomplete penetrance (approximately 66%), meaning some carriers of pathogenic variants may remain asymptomatic throughout life, and high phenotypic variability. The spectrum of clinical manifestations is broad, ranging from asymptomatic carriers to severe central nervous system dysfunction, including various types of seizures and neurodevelopmental delays. This clinical diversity arises primarily from the loss-of-function nature of most DEPDC5 gene mutations. Combined with the mechanisms of germline and somatic mutations described above, along with other genetic modification mechanisms, these factors collectively contribute to the complex genetic-phenotypic diversity observed in DEPDC5 disease (Baulac et al., 2016; Swaminathan et al., 2018).

In recent years, the spectrum of disease phenotypes caused by DEPDC5 gene variants has gradually expanded. Early studies primarily associated it with familial focal epilepsy, and this gene is also considered one of the most common causative genes for this type of epilepsy, accounting for approximately 12–37% of cases (Baulac et al., 2015). However, mounting research evidence indicates that DEPDC5 gene mutations can also lead to a broader clinical spectrum, including developmental epileptic encephalopathies and febrile seizures (Horiuchi et al., 2024). Unlike multi-systemic conditions like tuberous sclerosis, the phenotypes caused by DEPDC5 mutations typically involve only the nervous system, particularly affecting dendritic and spinal development. This results in neurosystem-specific symptoms, suggesting the gene has unique functional roles within the nervous system (Steinlein, 2014; Bisulli et al., 2021). Furthermore, while isolated studies have reported associations between DEPDC5 mutations and certain tumors (e.g., glioblastoma, ovarian cancer, hepatocellular carcinoma), no definitive evidence currently exists demonstrating a direct functional contribution of these mutations to carcinogenesis. The causal relationship and molecular mechanisms underlying tumorigenesis remain to be further explored (Parthasarathy et al., 2021).

This study reports the clinical and genotype characteristics of eight children with DEPDC5-related epilepsy diagnosed in the Department of Neurology at Hebei Children’s Hospital between April 2020 and November 2024. The aim is to expand understanding of the phenotypic diversity associated with DEPDC5 gene variants and their impact on epilepsy. Analysis of these clinical cases further explores the clinical manifestations and pathogenic mechanisms of DEPDC5 mutations across different phenotypes, strengthening our understanding of the relationship between this gene mutation and neurological disorders.

## 2 Objects and methods

### 2.1 Objects

The clinical data of eight children with DEPDC5 gene variant-associated epilepsy were retrospectively collected from the Department of Neurology at Hebei Provincial Children’s Hospital between April 2020 and November 2024. The study protocol was approved by the Ethics Committee of Hebei Provincial Children’s Hospital, and written informed consent was obtained from the parents or legal guardians of all participating children.

### 2.2 Methods

#### 2.2.1 Genetic testing

Genetic testing was performed using whole exome sequencing (WES) followed by Sanger sequencing to validate the identified mutation sites and determine their origin. The pathogenicity of the mutations was evaluated in accordance with the guidelines established by the American College of Medical Genetics and Genomics (ACMG). Based on these analyses, the diagnosis of DEPDC5 gene variant-associated epilepsy was confirmed in all cases, establishing a clear genotype–phenotype correlation.

#### 2.2.2 Clinical data collection

Clinical information was obtained by reviewing electronic medical records from both outpatient and inpatient visits. Data included the child’s gender, age at epilepsy onset, seizure types, personal medical history, family history, and results of ancillary tests such as electroencephalogram (EEG), cranial magnetic resonance imaging (MRI), blood and urine metabolic screening, and genetic reports. Treatment details, including antiepileptic medications used, were also recorded. Prognostic data were gathered through outpatient follow-ups, re-hospitalization records, and telephone interviews, documenting follow-up duration, seizure frequency, medication adjustments, and developmental progress.

#### 2.2.3 Diagnostic criteria

Epilepsy and epileptic syndromes were diagnosed and classified in accordance with the most recent diagnostic frameworks published by the International League Against Epilepsy (ILAE) in 2017 and 2022.

Observation indicators and evaluation criteria (observation period: 1 year after initiating antiseizure medicationstherapy):

Complete control: no seizures occurred during the observation period.
Significantly effective: seizure frequency decreased by more than 75% compared to the year prior to treatment.
Effective: seizure frequency decreased by 50–75% compared to the year prior to treatment.
Ineffective: seizure frequency showed no significant reduction or worsened compared to the year prior to treatment.

The overall effective rate was calculated as: (complete control + significantly effective + effective)/total number of cases × 100%.

### 2.3 Statistical methods

Data were statistically analyzed and organized into tables using Microsoft Excel software.

## 3 Results

### 3.1 Genetic results

**Figure 1:**
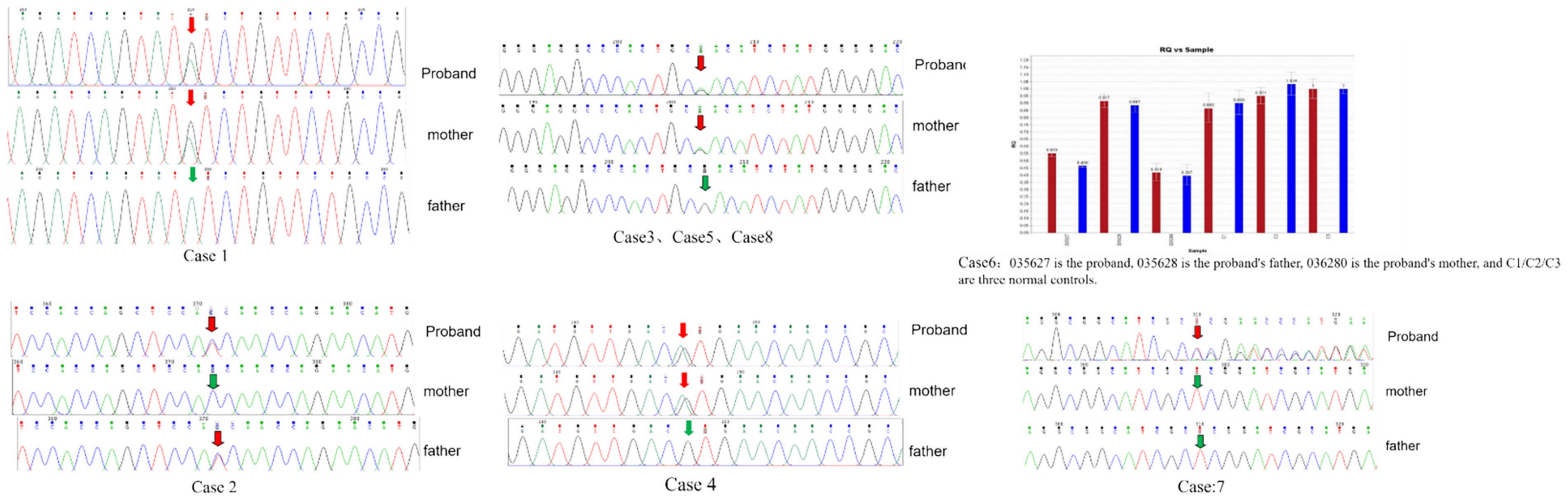
Sanger sequencing of gene mutations in 8 pediatric patients.

Among the eight children with heterozygous mutations in the DEPDC5 gene, six exhibited missense mutations (c.2773A > G/p.I1925V, c.2899G > A/p.D967N, c.4601C > T/p.T1534I, and c.4078G > A/p.V1360M), with three cases involving c.2899G > A/p.D967N. Additionally, one case presented a frameshift mutation (c.3004del/p.S1002Rfs*4), and one case displayed a large segment deletion (seq[GRCh37]22q12.2q12.3). Genetic analysis identified one *de novo* mutation and seven inherited variants, including one of paternal origin and six of maternal origin. A review of the human mutation database revealed that two variants had been previously reported (c.2899G > A/p.D967N and c.4078G > A/p.V1360M), while four variant loci were novel. These four novel variants were classified as variants of uncertain significance (VUS) according to the American College of Medical Genetics and Genomics (ACMG) guidelines but were predicted to be deleterious based on bioinformatics software analysis. The detailed Sanger diagram of mutation sites is shown in Figure 1.

### 3.2 Clinical results

Among the eight children with heterozygous variants in the DEPDC5 gene, three were female and five were male. The age at epilepsy diagnosis ranged from 1 year and 4 months to 9 years and 3 months. Two children had a family history of epilepsy and febrile seizures. In one case, the father, brother, and sister had a history of febrile seizures during early childhood, and the father carried a heterozygous DEPDC5 variant (c.4601C > T/p.T1534I), which was consistent with the child’s variant locus. In another case, the mother had a history of seizures and carried a heterozygous DEPDC5 variant (c.2899G > A/p.D967N), matching the child’s variant locus (Table 1).

**Table 1:** Clinical characteristics of children with DEPDC5 gene mutations.

Case	Mutation site	Mutation type	Source	Diagnosis	Age of onset	MRI	Development	Anti-seizure medications	Control situation
1	c.2773A > G/p.I1925V	Missense	Mother	Seizure	4 years 2 months	Normal	Normal	LEV	25% reduction
2	c.4601C > T/ p.T1534I	Missense	Father	Seizure	1 year 9 months	Normal	Normal	LEV	Seizure-free
3	c.2899G > A/p.D967N	Missense	Mother	SeLECTS	9 years 3 months	Normal	Normal	LEV	Seizure-free
4	c.4078G > A/p.V1360M	Missense	Mother	SeLECTS	6 years 10 months	Normal	Normal	LEV	Seizure-free
5	c.2899G > A/ p.D967N	Missense	Mother	LGS	2 years	Delayed myelination	Delayed	LEV, PB	25% reduction
6	seq[GRCh37]22q12.2q12.3	Deletion	Mother	LGS	1 year 8 months	Left frontoparieto-occipital microglossal malformation	Delayed	PB	25% reduction
7	c.3004del/p.S1002Rfs*4	Code Shift	Neonatal	LGS	1 year 4 months	Delayed myelination	Delayed	PB, VPA, OXC, PER	Not valid
8	c.2899G > A/ p.D967N	Missense	Mother	LGS	3 score	Delayed myelination	Delayed	LEV	Not valid

SeLECTS is self-limited epilepsy with central temporal spikes; LGS is Lennox–Gastaut syndrome; LEV is levetiracetam; PB is phenobarbital; VPA is valproate; OXC is oxcarbazepine; and PER is pirempanel.

Clinically, five children exhibited generalized tonic–clonic seizures, one had tonic seizures, and two pediatric patients exhibited two seizure types: tonic seizures and atonic seizures. At the last follow-up, the children’s ages ranged from 2 years and 6 months to 11 years and 4 months. Four children showed global developmental delay (age range: 1 year and 4 months to 3 years), while the remaining four demonstrated normal developmental progression.

### 3.3 Ancillary examinations

**Figure 2:**
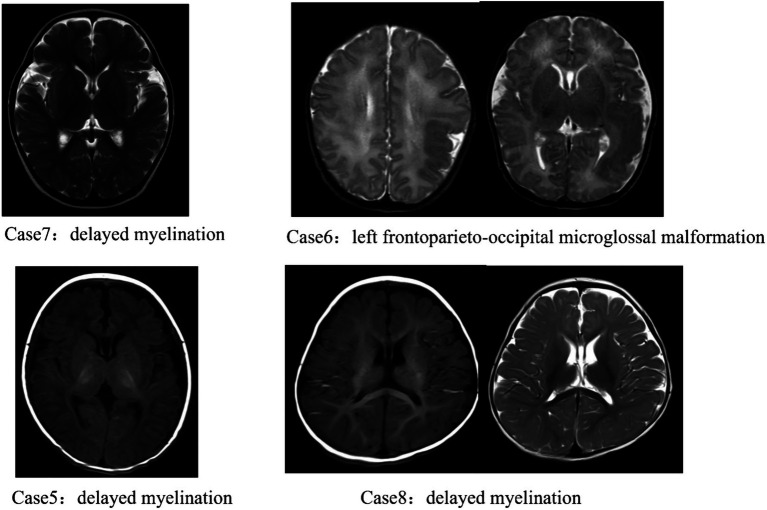
Abnormal cranial magnetic resonance imaging results of 4 pediatric patients.

All children underwent video EEG monitoring for at least 4 h. Abnormal discharges were observed in all eight cases. One case exhibited widespread 2–3 Hz delta slow wave activity intermittently throughout the recording. Another case showed a small number of medium-amplitude suspected spike waves in the right hemisphere during sleep. One case demonstrated predominantly medium- to high-amplitude 1–2 Hz spike-and-slow-wave rhythmic paroxysms in the left hemisphere during sleep, lasting 5–6 s. Two cases displayed bilateral Rolandic spike-and-slow-wave discharges during both wakefulness and sleep. One case presented multifocal (predominantly bilateral anterior head) spike-and-slow-wave discharges during wakefulness and sleep. Another case showed left anterior head spike-and-slow-wave discharges during wakefulness and sleep, while one case exhibited right posterior head spike-and-slow-wave discharges during wakefulness. Additionally, one case had left anterior head spike-and-slow-wave discharges during sleep. A focal seizure was captured during monitoring in one case. See Table 2 for details. Among the eight children, cranial magnetic resonance imaging (MRI) revealed abnormalities in four cases, while the remaining four showed no structural anomalies. The observed abnormalities included delayed myelination, hyaline septal cavities, and widening of the frontal-temporal extracerebral spaces in three cases, as well as left frontoparieto-occipital polymicrogyria malformation in one case. See Table 1 and Figure 2 for details.

**Table 2:** EEG results of 8 pediatric patients.

Case	EEG	Electroencephalogram after treatment
1	Sharp wave	Normal
2	Slow wave	Normal
3	Bilateral Rolandic zone spikes, spikes and slow waves	Normal
4	Bilateral Rolandic zone spikes, spikes and slow waves	Blinking left Rolandic area with spike waves, multiple spike waves, spike waves, spike slow waves/spike slow waves during each sleep period.
5	Multifocal spikes, spikes and slow waves	Loss to follow-up
6	Medium-high amplitude sharp slow waves	Loss to follow-up
7	Spike, spike slow wave	During wakefulness, left anterior head spine waves and slow spine waves are distributed, while during sleep, left anterior head spine waves and slow spine waves are distributed.
8	Spike, spike slow wave	normal

### 3.4 Diagnosis, treatment and prognosis

Two children, aged 9 years and 3 months and 6 years and 10 months, respectively, presented with generalized tonic–clonic seizures. Their EEGs revealed bilateral Rolandic spikes and slow waves, and both exhibited normal developmental milestones, leading to a diagnosis of Self-Limited Epilepsy with Centrotemporal Spikes (SeLECTS). Four children, with ages of onset ranging from 1 year and 4 months to 3 years, displayed seizure types including tonic seizures, catatonic seizures, and generalized tonic–clonic seizures. Their EEGs showed multifocal spikes and spike-and-slow-wave discharges, and all four exhibited developmental delay, resulting in a diagnosis of Lennox–Gastaut Syndrome (LGS). The remaining two cases met the diagnostic criteria for epilepsy but did not fulfill the criteria for a specific epilepsy syndrome.

At the last follow-up, the ages of the eight children ranged from 2 years and 6 months to 11 years and 4 months. Two cases showed poor response to medication, despite treatment with levetiracetam, sodium valproate, phenobarbital, oxcarbazepine, and perampanel, either as monotherapy or in combinations of up to three anti-seizure medications (ASM). In contrast, six cases achieved effective seizure control with pharmacologic intervention. Among these, three children achieved seizure freedom with levetiracetam monotherapy, one experienced a 25% reduction in seizure frequency with levetiracetam, one achieved a 25% reduction with a combination of levetiracetam and phenobarbital, and one showed a 25% reduction with phenobarbital alone.

## 4 Discussion

Familial focal epilepsy was the first epilepsy phenotype identified to be associated with DEPDC5 gene variants, and it has been widely reported in international studies. This phenotype primarily includes autosomal dominant sleep-related hyperkinetic epilepsy, familial medial temporal lobe epilepsy, and autosomal dominant epilepsy with auditory features (Horiuchi et al., 2024). In recent years, pathogenic variants in the DEPDC5 gene have been linked to a broad spectrum of epilepsy phenotypes, ranging from mild conditions such as febrile seizures and febrile seizure plus to severe developmental epileptic encephalopathies like West syndrome (Baldassari et al., 2019; Samanta, 2022). In this study, As shown in Table 1, eight children with DEPDC5 variants exhibited significant phenotypic heterogeneity. Two children were diagnosed with Self-Limited Epilepsy with Centrotemporal Spikes (SeLECTS), characterized by a later age of onset (6 to 9 years), favorable treatment response, and better prognosis. Four children were diagnosed with Lennox–Gastaut Syndrome (LGS), presenting with an earlier age of onset (1 year and 4 months to 3 years), global developmental delay, and poorer outcomes. The remaining two children met the diagnostic criteria for epilepsy but did not fulfill the criteria for a specific epileptic syndrome.

Previous studies have indicated that nonsense and splice-site mutations in the DEPDC5 gene are associated with severe developmental epileptic encephalopathy phenotypes, often accompanied by additional neurological disorders such as intellectual disability, migraines, autism spectrum disorders, and psychiatric conditions. In contrast, patients with frameshift or missense mutations typically exhibit a more favorable prognosis (Zhang et al., 2021). However, in this study, among the four children diagnosed with Lennox–Gastaut syndrome (LGS), the mutation types included two missense mutations (c.2899G > A/p.D967N), one frameshift mutation (c.3004del/p.S1002Rfs*4), and one large segment deletion (seq[GRCh37]22q12.2q12.3). All four children demonstrated global developmental delay and poor prognosis, with the child harboring the segment deletion exhibiting particularly severe refractory epilepsy. These findings suggest that missense mutations, frameshift mutations, and large segment deletions in the DEPDC5 gene can lead to severe epileptic phenotypes. Notably, segment deletion mutations may be associated with more severe clinical manifestations. This underscores the importance of early clinical recognition, diagnosis, and intervention for such cases.

In this study, three children were identified as carrying the c.2899G > A/p.D967N mutation locus, which has previously been reported in only one case presenting with a focal epilepsy phenotype (Tsai et al., 2017). Notably, the three children in our study exhibited diverse phenotypes: two were diagnosed with Lennox–Gastaut syndrome (LGS), and one with Self-Limited Epilepsy with Centrotemporal Spikes (SeLECTS), with ages of onset ranging from early childhood to school age. According to Marsan et al., homozygous DEPDC5 mutant rats exhibited growth delay and eventual mortality, whereas heterozygous mutants developed normally but experienced spontaneous seizures and survived (Marsan et al., 2016). Although the three children in our study were heterozygous for the c.2899G > A/p.D967N mutation, their phenotypic differences were significant, potentially due to epistatic heterogeneity. Furthermore, we observed that children carrying the c.2899G > A/p.D967N mutation locus may present with benign epilepsy, such as SeLECTS, if the onset occurs in early childhood. In contrast, those with school-age onset were more likely to develop severe epileptic phenotypes, such as LGS. This suggests that the age of onset may significantly influence the phenotypic expression of the c.2899G > A/p.D967N mutation locus in the DEPDC5 gene. While the age of onset may provide preliminary predictive value for the severity of phenotypes, other factors such as medication duration and time to diagnosis must also be considered.

In this study, children carrying the c.4601C > T/p.T1534I, c.4078G > A/p.V1360M, and c.2773A > G/p.I1925V mutation loci exhibited better treatment outcomes and prognosis for epilepsy. In contrast, the two children carrying the c.2899G > A/p.D967N mutation locus presented with distinct phenotypes: one with benign self-limited epilepsy and the other with refractory epilepsy. A previous study by Liu et al. suggested that heterozygous missense mutations in the DEPDC5 gene, particularly those located in regions distant from its encoded protein complex, may result in milder epileptic phenotypes such as febrile seizures or febrile seizure plus (Liu et al., 2020). In our study, further analysis of genetic subregions in five children revealed that the mutation sites in three children with better epilepsy control were located in the CDS28, CDS29, CDS42, and CDS38 subregions, respectively. Conversely, the mutation site in one child with refractory epilepsy was located in the CSD29 subregion. These findings partially align with previous studies but suggest that the CSD29 subregion of the DEPDC5 gene may be associated with both benign and refractory epilepsy. This phenotypic variability could be attributed to differences in the degree to which the mutations impair the function of the protein complex. Given the limited sample size, further cases will be collected, and protein function analyses will be conducted to validate these hypotheses.

Abnormal brain structure is a significant feature of epilepsy phenotypes associated with DEPDC5 gene variants. In this study, one child carrying a large segmental deletion exhibited left frontoparieto-occipital polymicrogyria malformation, while three children with missense or frameshift mutations showed delayed myelination. The remaining 50% of the children displayed no obvious structural brain abnormalities. According to previous studies, this variability may be linked to the mutation rate in the DEPDC5 gene and the presence or absence of concomitant somatic mutations in other genes (Picard et al., 2014; Baulac et al., 2015). Additionally, several studies have demonstrated that surgical intervention can effectively control seizures in children with epilepsy and structural brain abnormalities (Baldassari et al., 2019; Sanders et al., 2019). In this study, one child treated with phenobarbital achieved a 25% reduction in seizure frequency. If seizures are not fully controlled during subsequent follow-up, further evaluation and consideration of surgical treatment will be recommended.

Studies have indicated that the majority of patients with DEPDC5 gene variant-associated epilepsy exhibit drug-refractory epilepsy (Sanders et al., 2019). However, recent international case reports have demonstrated the efficacy of carbamazepine in managing focal epilepsy phenotypes (Mulkerrin and Hennessy, 2024). A domestic study by Chen Li-Qing et al. revealed that aminocaproic acid effectively controlled seizures in West syndrome caused by DEPDC5 gene mutations (Chen et al., 2019). Additionally, a study involving 20 cases of DEPDC5-related epilepsy phenotypes found that medications such as lamotrigine and sodium valproate achieved favorable therapeutic outcomes in some patients (Liu et al., 2021). For refractory epilepsy phenotypes linked to DEPDC5 gene variants, interventions such as the ketogenic diet and lacosamide have also been shown to significantly reduce seizure frequency (Tokorodani et al., 2024; Ververi et al., 2023).

Mechanistically, the DEPDC5 gene contributes to epilepsy development by regulating the mTOR pathway. Consequently, anti-seizure medications that inhibit the mTOR pathway, such as rapamycin, may theoretically offer the most effective treatment. However, the clinical application of rapamycin has been limited due to its associated side effects (Myers and Scheffer, 2017). In this study, two children with Lennox–Gastaut syndrome (LGS) treated with phenobarbital and levetiracetam experienced a 25% reduction in seizure frequency. For the other two children with LGS who showed poor response to multiple antiseizure medicationss, including levetiracetam and sodium valproate, alternative treatment options such as aminocaproic acid, the ketogenic diet, or lacosamide could be considered to further enhance therapeutic efficacy.

In this study, we describe the clinical phenotypes and genetic characteristics of eight children with DEPDC5 gene variant-associated epilepsy, including the identification of four *de novo* mutations. The results demonstrate that children carrying different mutation types or even the same mutation locus exhibited diverse clinical phenotypes. Building on previous studies, we hypothesize that the age of onset and genetic subtype may contribute to this phenotypic heterogeneity. Furthermore, we observed that children with missense mutations, frameshift mutations, and large segment deletions tended to present with severe phenotypes and comorbidities. Notably, Levetiracetam and phenobarbital can reduce seizure frequency in some individual patients. These findings provide valuable insights for clinicians in selecting treatment strategies and evaluating prognosis for children with DEPDC5 gene variant-associated epilepsy.

However, this study has several limitations: (1) The small sample size and absence of a control group may limit the reliability of conclusions related to anti-seizure medications efficacy; (2) Some children were too young for comprehensive developmental assessment, relying primarily on indicators such as gross motor function, which may introduce some degree of bias; and (3) The follow-up period was relatively short. Future studies should extend the follow-up duration to better observe seizure control and developmental progress following treatment adjustments.

## Data Availability

The original contributions presented in the study are included in the article/supplementary material, further inquiries can be directed to the corresponding authors.
